# Case of Disease Observed in the Hospitals of Paris

**Published:** 1853-06

**Authors:** Elkanah Williams

**Affiliations:** Cincinnati; Paris


ART. III.—*Cases of Disease observed in the Hospitals of Paris* By ELKANAH WILLIAMS. M. D., of Cincinnati.

CASE 1—*Inguinial Hernia, complicated with protrusion of the bladder.*—About the middle of January, a man entered the service of M. Roux, at Hotel Dieu, with strangulated hernia. It was congenital inguinal hernia of the right side. The patient previous to the age of 21 years had never worn any apparatus to keep it reduced. After that time he procured some kind of a bandage which, however, did not retain the intestines in the abdomen. For four days previous to entering the hospital, he had been laboring under urgent symptoms of strangulation—repeated attempts at taxis having failed. When M. Roux first saw him, the tumor was very large, and the patient in a very grave condition; so he at once decided upon an operation, considering the case one of ordinary hernia. The first part of the operation was very simple—soon he reached the strangulated portion of intestine, divided the stricture and returned it. In the lower and internal part of the sac, however, was then seen a tumor of considerable size with a smooth and polished surface, and some small granular deposits of adipose tissue, which he took for the epiploon. In passing the finger around the base of it, there seemed to be no adhesions. He attempted to reduce it and, in part, succeeded. As soon as the finger abandoned the reduced portion it at once reprotruded. More fully satisfied than ever, that it was omentum, he concluded to *excise* it. He commenced by cautiously *paring* off a small part at a time; at length an opening was made through which suddenly gushed a quantity of *urine!* Then for the first time he saw that it was a cystocele and that he had opened the bladder. He immediately closed up the small wound he had made, and applied the usual dressings. During the day the patient had one or two evacuations from the bowels; still the symptoms of strangulation persisted, and the patient died twenty-six hours after the operation. On post-mortem examination it was found that, at least, *half* the *bladder* protruded into the hernial sac. It seems most probable that the bladder had been thus protruded for many years by the side of the hernial portion of intestine, without giving rise to any symptoms. Indeed, M. Roux never once thought of the bladder till he had punctured it and saw the discharge of urine, and says it is the first case of hernia with this complication that he had ever seen. In an account of this operation which he published in the "Gazette des Hopitaux," he says—"While I am cheerful to avow my mistake, I must add that I do not think it had any influence on the result of the case." The extreme rarity of this complication of inguinal hernia and the mistaken diagnosis by this distinguished surgeon, invest the case with very great interest. M. Roux is one of the most beautiful operators that I have ever seen; besides he is a candid, honest, and exceedingly pleasant man. He has, perhaps, performed more capital operations than any surgeon in Europe.—Although over sixty years of age, his hand is perfectly steady, and he uses the knife with astonishing accuracy and rapidity.

Case 2.—*Operation for producing obliteration of the Vagina.*—A woman aged twenty-three, entered the hos pital Cochin, on the 12th ult.; in the service of M. Maisonneuve, five months ago, she was confined, and the labor was exceedingly difficult. At the end of five days, I believe, she was delivered of a dead fœtus, by means of the forceps. Severe inflammation and extensive sloughing followed; and she was left with an enormous vesico-vaginal and recto-vaginal fistula. The whole of the urine and feces passed out involuntarily through the vagina, than which no infirmity could be more horrible. The case was not remediable by any of the ordinary operations for vesico-vaginal fistulas. Some years ago, M. Vidal de Cassis suggested the idea of obliterating the vagina in cases of incurable fistulas of this sort. Several attempts at different times were made by cauterization, &c., &c., but without success. It seemed that surgery could not obliterate the vagina; although nature herself, sometimes, does it in spite of the surgeon. Some three years since M. Maisonneuve attempted it by the following operation. He incised the mucous membrane around the outlet of the vagina, dissected it up with the sub-mucous cellular tissue some 1½ or 2 inches along the vagina—so that he thus had as it were a loose sleeve of mucous membrane hanging loosely in this canal. He then attached the sides of the free end of this sleeve with a peculiar suture, pushed it up, as you would invaginate the finger of a glove, and then brought the denuded sides of the outlet of the vagina together by the quill suture passed deeply through the vulva. The parts united by the first intention—but unfortunately there was no outlet for the urine—the urethra having previously become obliterated. In order to produce an artificial urethra, to terminate in the rectum, so that she might have voluntary control over the discharge of the urine, he made a uncture through the perineum from within the sphincter ani to the bladder. The patient died from the effects of this puncture; but *died*, as the patients of French surgeons often do, *cured!* Encouraged by his success in that instance, he resolved to try it again in this case, and operated the 12th inst. After dissecting up the mucous coat as before, he united the outlet of the loose sleeve with a suture so arranged, that when this cul de sac, thus formed, was pushed up into the vagina its *mucous surface*, exclusively, presented upwards so as to come in contact with the fecal matters and urine. He then brought the denuded walls of the outlet of the vagina together with the quill suture—one quill placed antero-posteriorly along the base of the labiæ, leaving the urethra and meatus urinarius free.

May 17. Patient has rested comfortably since the operation; some feces have passed by the rectum. On removing the sutures, the wound partially opened, and the fecal matters passed again in considerable quantities by the vagina. Through a sound introduced into the bladder, the same mixture of urine and fecal matter passed. Hence it appears that the presence of *urine* in the *rectum* or *feces* in the *bladder* does not produce *serious irritation*, as this woman felt no inconvenience from it. By means of compresses and the bandage he confined the sides of the vaginal outlet firmly together after removing the sutures, and this treatment is still continued. Whether the operation will succeed fully or not, I can not say. I see nothing in the nature of the case to forbid it; I will give you the result at some future time. It is necessary to state that in this woman the whole of the basfond of the bladder had been destroyed by the sloughing, so that the opening between it and the vagina is enormous; likewise at least half of the anterior wall of rectum for 2 or 2½ inches is destroyed. The usual operation for vesico-vaginal fistula, by paring the edges and uniting by sutures, could only be effected by dissecting the bladder loose from the surrounding pelvic walls to a large extent. The contraction of the cicatrix thus formed around the bladder would inevitably tear open again the fistula even if it united favorably in the first place. Besides, the cavity of the bladder is very greatly contracted—not capable of holding, perhaps, over an ounce or two of urine. To unite the recto-vaginal fistula in the usual way, would be to produce an unmanageable stricture of the rectum. Hence no other course was left but to attempt the obliteration of the vagina, or leave the poor woman with the loathsome infirmity of the constant discharge of all the urine and feces by this outlet. If the operation succeeds, the woman will have control over the discharge of these excretions, and that is what she desires above all other considerations. This is a new and simple operation which may be susceptible of valuable application in such incurable cases.

Case 3.—*Chronic Farcy.*—On the 8th of April, 1853, there entered the Hospital of the Faculty a man aged 42, occupation, coach driver. For several days previous to the commencement of his disease, he had been employed in the stable of a veterinary surgeon, where there was a horse affected with *glanders.* On the 10th February, after a severe day's labor, he began to experience pain in the back and limbs, lassitude and headache, followed, in a few hours, by an attack of shivering, which was repeated several times in the course of the ensuing 24 hours. He, however, did not abandon his work. Some five days afterwards, this feeling of general "malaise" and loss of appetite persisting, he felt pain and soreness in the inferior external part of the left thigh, also, in the same leg, a few inches below the knee. A physician was consulted, who supposed the disease to be rheumatism, and ordered a small blister over each of the painful points; temporary relief only was the result of this application. In a few days acute pain was developed in the bottom of the left foot, which was so much aggravated by the pressure of walking that he was, for the first time, compelled to quit his work.

The sensation of extreme weariness, the general muscular soreness and loss of appetite still continued; worse one day and better the next, but never entirely disappearing. On the 1st March, the same kind of *circumscribed pain and tenderness* was felt in the calf of the right leg, the middle, anterior part of the thigh and on the outside of the corresponding arm. His sufferings and general indisposition augmenting from day to day, he was compelled to enter the hospital for surgical aid on the 8th April. By a slight examination an abscess was detected in the bottom of the left foot, which, being punctured, gave issue to a considerable quantity of thin sanious pus. The following day M. Nélaton, the surgeon in chief of the hospital, discovered five other abscesses, occupying the points designated by the patient as being the seats of the previous sufferings. The skin immediately over these purulent collections was perfectly natural, and pressure produced only slight suffering; in short, they presented all the appearances of chronic or cold abscesses. The fact of the patient's having been exposed in the stable to the contagion of glanders, combined with the history and actual condition of the case, induced M. Nélaton to pronounce the disease chronic farcy. To render the diagnosis, however, more certain, he inoculated a venerable old ass with some of the pus of one of these collections. But the unfortunate animal *died of famine* before sufficient time had elapsed for the development of the disease, and thus was deprived of the honor of falling a *martyr to science!* No other experiment was made.

As to treatment, the patient was put upon nourishing broths, wine and tonics, of which the sulphate of quinine was the most important. The abscesses were punctured one by one, at intervals of one or two days, with a narrow bistoury, and injected with tincture of iodine.

April 13. Patient's general sufferings somewhat diminished; he sleeps well, and has not the appearance of being very profoundly ill; appetite still wanting, and there is some pain in the region of the abscesses on the outside of the right leg.

April 15. Constitutional symptoms rather better. All the abscesses but one have been punctured, and discharged a thin sanious pus; are daily injected with iodine. General treatment same, with addition of tincture of aconite in small doses.

April 18. Yesterday evening erysipelas attacked the right leg and foot, and he has suffered considerably during past night. The foot and leg, as far as knee, are somewhat swollen, and œdematous, integuments red. A sero-purulent fluid continues to be discharged from the cavities of all the abscesses, in considerable quantities.

April 22. Elevated position and emollients have relieved the erysipelas. Condition of patient in other respects more unfavorable.

April 27. But little change in the case; no new abscesses have appeared, but the original ones continue to discharge large quantities of unhealthy pus. It seems impossible to produce adhesive inflammation and obliteration of the sacs by the injections.

May 2. This morning the patient is much exhausted; features contracted and anxious; tongue dry, with a brown coat in the middle; some sordes upon the teeth; pulse extremely frequent and feeble; skin dry; appetite entirely gone. He complains of very great *soreness of the throat, and his voice is reduced to a whisper.* Several new abscesses have appeared upon the arms. On the back of each hand is a circumscribed swelling, with a livid redness and severe pain.

May 3. 9 A. M. Inflammation of throat greatly increased; mouth very dry; lips, tongue and teeth covered with brown sordes; extreme exhaustion; great difficulty of breathing, with blueness of face and surface generally, indicating failure of the function of aeration and a great alteration of the blood. *Voice extinct.*

His condition rapidly growing worse, the patient died at 4 P. M., on the same day, having retained his intellectual powers till the last.

Post mortem 24 hours after death. In addition to the abscesses detected before death, two others only were discovered—one in the substance of the triceps extensor cruris muscle, the other in the triceps extensor cubiti. The superficial veins of the arms exhibited numerous dilatations, but contained no clots or other evidence of phlebitis. Mucous membrane of the nares slightly congested; that of posterior part of pharynx and of the larynx exhibited extensive inflammatory lesions; *epiglottis riddled with ulcers, and vocal chords destroyed.* Upper part of trachea congested. No alteration of the lungs, except some congestion and slight increase of density of the middle lobe of the right. No *metastatic abscesses* were found in any of the viscera, nor sero-purulent effusions in the joints, which is often the case when death results from *phlebitis* and the so called purulent *absorption.* Blood *fluid, venous* and containing a large quantity of serum. At several points the skin of the chest exhibited circumscribed engorged bluish spots, somewhat thickened, indicating the *forming stage of pustules.* Doubtless, if the patient had lived a few days longer, the mucous membrane of the nares would have been invaded, and he would have had the discharge of muco-pus from the nose, which is peculiar to the glanders. In chronic farcy the patient is nearly always carried off by an acute attack of glanders. The duration of the disease varies from two months to two or three years, the average being about eight months. The communication of the glanders from the lower animals to the human species and from man to man, although long known, was never, I believe, particularly commented on by medical writers till since the beginning of the present century; and it is only a few years since it was fully established by observation, and especially by experiments on the inferior animals, that the *glanders* and the farcy are *modifications* of the *same disease*, acknowledging the *same specific virus as their common cause.* Inoculation with the muco-pus of glanders develops, sometimes, the same disease, at others the farcy, acute or chronic. Moreover, inoculation with the pus of the abscesses of the farcy gives rise indifferently either to the glanders or the farcy. Observation has proved that this reciprocal production of each other is true of these maladies as they occur in man. The case above cited is an identical example of *chronic farcy*, contracted from the *glanders* in a horse. The termination of the case by the supervention of an attack of acute glanders, renders the proof of the common origin and close connection of these two diseases still more positive. Both affections are communicable, either by contagion or infection, from the inferior animals to the human species, and from man to man. This patient seems to have received the poison by infection, through the medium of the air, as he had no abrasion or wound of the skin; besides this, the *general* symptoms were revealed *first*, the *local secondarily.* When contracted by contagion the reverse in the order of the phenomena is true—first the *local lesion*, then *constitutional disturbance*, as in the case of a dissecting wound. In farcy by inoculation, the lymphatic vessels and veins leading from the wound are first invaded, followed by engorgement and abscesses of the lymphatic ganglions, and finally extension of the inflammation to the subcutaneous cellular tissue. At length general symptoms of the gravest character ensue, indicating serious changes in the vital properties of the solids and fluids.

When the poison is introduced into the system by infection, its first effects are loss of appetite, lassitude, shiverings, general aching and soreness in the muscles and joints

Sooner or later the patient experiences pains in certain circumscribed points of the extremities, with slight soft swelling and the rapid formation of pus and abscesses, which very rarely heal after their evacuation, in consequence of the insusceptibility of the tissues to take on adhesive inflammation.

The prognosis of chronic farcy is exceedingly grave, although it is not so invariably fatal as the acute form or the genuine glanders. It is said that the horse rarely *fully recovers* from an attack of this disease. He remains feeble and "short winded," and is very liable to chronic pleurisy and hydrothorax. As to their gravity and the symptoms, especially in the *advanced stages*, there are many striking analogies between glanders, farcy, malignant pustule, phlebitis, dissecting wound, some forms of erysipelas, and the so called purulent absorption. In all of them there is a profound alteration in the composition of the blood, producing great exhaustion of the vital forces, with strong disposition to suppuration, the formation of abscesses, external and internal, and, often, extensive gangrene. As to *identity of cause*, farcy and glanders stand in the same relation to each other as do malignant pustule and the dreadful disease described by French writers under the name of "charbon."

The points of peculiar interest in this case are—

1st. Its cause—the virus of glanders received from the horse.

2nd. The manner of its introduction into the system—by infection.

3rd. The duration of the attack—nearly three months.

4th. The transitory local pains about the joints mistaken at first and treated for *rheumatism.*

5th. The apparently mild character of the disease and its frequent amelioration during the first two months, which would have induced a surgeon, unacquainted with the serious character of the affection, to have pronounced a favorable prognosis.

6th. The final termination of the case by an attack of acute glanders.

Paris, May 23, 1853.

